# Weakly supervised anatomical feature learning for cross-dataset ejection fraction estimation from echocardiography videos

**DOI:** 10.1186/s12880-026-02781-7

**Published:** 2026-09-16

**Authors:** Adrian Krenzer, Viktoria Wieser, Tobias Friedetzki

**Affiliations:** Department of Artificial Intelligence in Medical Applications, Julius-Maximilians University of Würzburg, Sanderring 2, 97070 Würzburg, Germany

**Keywords:** Echocardiography, Ejection fraction, Weak supervision, Annotation-efficient learning, Medical image analysis, Cross-dataset evaluation, Contrastive learning, Feature extraction

## Abstract

**Background:**

Ejection fraction (EF) is a central measure of cardiac function, but echocardiographic EF assessment remains reader-dependent and sensitive to acquisition quality. Deep learning can automate EF estimation, yet performance measured on a single development dataset may not transfer to data acquired under different imaging and annotation conventions. We investigated whether anatomically constrained weakly supervised learning improves cross-dataset EF estimation.

**Methods:**

We propose CAFEx, a contrastive-augmented feature extraction pipeline that combines left ventricular segmentation, echocardiography-specific augmentation, mask-derived anatomical features, and temporal EF regression. The model was trained on EchoNet-Dynamic using video-level EF labels with limited dense annotations for the anatomical component. External evaluation was performed on CAMUS after image harmonization and recalculation of an apical-four-chamber monoplane EF endpoint. Performance was compared with reproduced segmentation-based, direct video-regression, graph-based, transformer-based, and feature-extraction baselines using Dice score, mean absolute error, and coefficient of determination.

**Results:**

In the harmonized train-on-EchoNet/test-on-CAMUS benchmark, anatomically constrained models degraded less than direct video-regression baselines. CAFEx achieved 90.73% Dice on CAMUS and improved CAMUS EF prediction over the strongest reproduced feature-extraction baseline, increasing $R^2$ from 0.38 to 0.54 and reducing mean absolute EF error from 7.89 to 6.70 percentage points. Poor-quality videos and extreme EF values remained challenging.

**Conclusions:**

Weakly supervised EF estimation can benefit from an anatomical bottleneck learned with limited dense annotation. The findings support controlled external validation under explicit image and target harmonization, while further prospective validation, calibration, and quality-control mechanisms are needed before clinical deployment.

## Introduction

Two-dimensional echocardiography is among the most widely used cardiac imaging modalities because it is accessible, inexpensive, fast, portable, and non-invasive. A central measurement derived from echocardiography is the left ventricular ejection fraction (EF), which quantifies the fraction of blood expelled from the left ventricle between end-diastole (ED) and end-systole (ES). EF is used for diagnosis, risk stratification, treatment selection, and longitudinal monitoring in cardiovascular disease. Reduced EF may indicate left ventricular remodeling, systolic dysfunction, myocardial infarction, or valvular heart disease [1, 2]. In heart failure, EF has a direct role in clinical decision making because therapeutic pathways differ between heart failure with reduced and preserved EF [3], and lower EF is associated with higher mortality [4].

Despite its central role, EF estimation from routine echocardiography is not a purely mechanical measurement. The commonly used biplane or monoplane Simpson procedure requires the clinician to identify the ED and ES phases, delineate the endocardial contour, place or infer the long-axis reference, and convert the resulting geometry into a volume estimate [5, 6]. Each of these steps is affected by acoustic shadowing, foreshortening, trabeculation, limited field of view, and reader-dependent interpretation of the basal contour. The practical consequence is that EF measurements exhibit inter- and intra-observer variability, particularly in borderline cases and in videos with reduced image quality [1, 7]. Automated learning-based methods therefore address a clinically relevant problem, but their evaluation should consider not only average accuracy in a benchmark dataset, but also the extent to which performance changes when acquisition conditions, annotation conventions, and patient distributions differ.

Deep learning has produced promising results for left ventricular segmentation and EF estimation. Convolutional segmentation networks, including U-Net-like and DeepLab-based architectures, learn endocardial localization from frame-level masks or tracings [7–10]. More recent EF estimation systems include direct video regression with attention or transformer backbones [11–13], graph neural networks over frame or anatomical features [14], and pipelines that extract explicit geometric descriptors from predicted masks [15]. Self-supervised, semi-supervised, and contrastive learning have also been introduced to reduce the dependence on dense manual annotation [16–19]. These developments indicate that the field is moving from pure segmentation accuracy towards representation learning under realistic supervision constraints.

The present work is motivated by this annotation-efficiency perspective. In a practical EF pipeline that uses routinely available clinical data, the cheapest and most widely available label is the video-level EF measurement already produced during routine interpretation. Dense contours, pixel-wise masks, and curated ED/ES frame labels are substantially more expensive and are usually available only for limited research cohorts. A model that depends on such dense labels at the scale of large retrospective datasets may therefore require additional adaptation when applied to new sites. Conversely, a model trained only by video-level supervision may learn shortcuts related to the dataset, scanner, or acquisition protocol rather than ventricular mechanics. This tension motivates an intermediate strategy: use limited dense annotations to learn an anatomical representation, but keep the EF regressor weakly supervised by the video-level EF label.

We study this strategy using EchoNet-Dynamic and CAMUS. EchoNet-Dynamic provides a large-scale echocardiography video benchmark with EF labels and sparse frame-level tracings, whereas CAMUS provides a smaller independent cohort with dense labels and a different target convention [7, 20–22]. This setting is useful because it separates in-distribution performance from performance on a harmonized external cohort. If a model performs well on EchoNet-Dynamic but performs poorly on CAMUS after target harmonization, the result suggests that the model has not learned a representation that transfers well in this specific evaluation of ventricular function. The goal is therefore not simply to maximize in-distribution accuracy, but to identify which form of supervision and representation is less sensitive to the evaluated dataset shift.

The main contributions of this paper are summarized as follows:

1. We formulate EF estimation as a weakly supervised video-level regression task using only EF labels, with dense annotations used selectively for anatomical feature learning.
2. We evaluate this setup in a train-on-EchoNet-Dynamic/test-on-CAMUS benchmark, where strong in-distribution performance does not fully transfer while anatomically guided features remain informative.
3. We present CAFEx, a contrastive-augmented pipeline combining pretraining, augmentation, mask-based feature extraction, and temporal modeling, improving $R^2$ from 0.38 to 0.54 and reducing MAE from 7.89% to 6.70% on CAMUS.

Taken together, these contributions suggest that recognition quality in echocardiographic EF estimation can benefit from using dense annotation strategically rather than scaling dense annotation to every training sample. The evidence is specific to the evaluated train-on-EchoNet/test-on-CAMUS setting and should be interpreted as support for further external validation rather than as proof of universal generalization.

## Related work

### Deep learning for echocardiographic segmentation

Left ventricular segmentation is the most common intermediate task in automated EF estimation because it closely matches the clinical workflow. Early deep learning systems for medical image segmentation were dominated by encoder–decoder convolutional networks, especially U-Net variants, which became attractive for biomedical applications because they combine localization and contextual abstraction in a data-efficient manner [8, 9]. DeepLab-style models extend this principle through atrous convolutions and multi-scale context aggregation [10]. In echocardiography, such models are frequently trained on ED and ES frames and then used to recover ventricular geometry for EF estimation [7, 23–25]. Their strength is interpretability: a predicted mask can be inspected, corrected, or converted into clinically meaningful measurements. Their weakness is the need for dense or contour-based supervision, which is expensive and sensitive to reader variability.

The importance of segmentation quality is particularly pronounced for EF estimation. Small contour errors at the base or apex can alter the estimated ventricular volume, especially at ED where the chamber is larger and the boundary is often less sharply defined. Moreover, frame-wise segmentation accuracy does not fully determine EF accuracy, because EF depends on the difference between EDV and ESV. A model may have a high mean Dice score and still produce biased EF values if errors are systematic across cardiac phases. This distinction motivates evaluating segmentation and EF prediction jointly rather than treating segmentation as an isolated endpoint.

### Direct video regression and temporal modeling

Direct video regression models attempt to learn EF from raw or weakly processed echocardiography videos. Such models avoid explicit feature engineering and can, in principle, exploit the entire cardiac cycle. Recurrent neural networks and long short-term memory networks provide one route to temporal aggregation, whereas transformer-based architectures model long-range temporal dependencies through attention [12, 26]. EchoCoTr and related models use modern video backbones and transformer components to predict EF directly from sequences [11, 13]. Graph-based models, such as EchoGNN, represent frames or anatomical entities as nodes and learn relations across time or structure [14]. Multistream and multitask approaches provide a related alternative by combining complementary views, frame-level information, or structural cues with EF prediction [27]. These approaches are attractive because they reduce manual design or jointly exploit several sources of information, but they also enlarge the hypothesis space. Without an explicit anatomical constraint, the model may associate EF with dataset-specific image statistics, sequence length, scanner characteristics, or acquisition artifacts.

The risk of shortcut learning is not unique to echocardiography, but it is amplified in ultrasound because the appearance of the same anatomy can vary markedly with probe position, gain, shadowing, and operator skill. A direct regressor trained on a single dataset may learn representations that are predictive under the source distribution yet unstable under external validation. This is why cross-dataset evaluation is a critical complement to conventional train/validation/test splits drawn from the same acquisition protocol.

### Weak, semi-supervised, and contrastive learning

Annotation-efficient learning has become central in medical image analysis. Weak supervision uses coarse labels, such as image-level or video-level labels, rather than dense pixel-wise annotations [28]. Semi-supervised learning combines a small labeled subset with larger unlabeled data, often through consistency regularization, pseudo-labeling, or teacher–student training [17]. Self-supervised and contrastive learning learn representations from pretext tasks or instance discrimination before task-specific fine-tuning [16, 18]. These methods are especially relevant for medical imaging because unlabeled images are often abundant, while expert annotations are expensive and time-consuming.

Recent related work has connected these ideas to recognition quality and domain generalization in medical imaging. Zhang et al. proposed a pyramid pixel context adaptation network that combines multiscale contextual modeling with supervised contrastive learning for medical image classification [29]. Li et al. proposed FreeSDG, a frequency-mixed single-source domain generalization method for medical image segmentation that uses frequency-domain augmentation and self-supervision to improve performance on unseen domains [30]. These studies are methodologically related to CAFEx through their use of representation learning and robustness-oriented augmentation, but CAFEx focuses on echocardiographic EF estimation through an LV-mask bottleneck and temporal anatomical features.

In the context of EF estimation, the phrase “weak supervision” has a concrete interpretation. A routine echocardiography study commonly contains a video-level EF measurement, but it rarely contains a dense mask for every frame. Thus, EF regression can be supervised by a global label, while segmentation supervision is available only for a limited number of frames or patients. This situation differs from fully supervised segmentation-based pipelines, in which the dense mask is the primary training signal, and from pure direct regression, in which the model receives no anatomical guidance. CAFEx occupies the middle ground: dense annotations are used to learn a reusable anatomical bottleneck, but the final EF task is supervised by video-level labels. In this sense, the method is annotation-efficient rather than annotation-free.

### External validation and dataset harmonization

External validation is important for clinical machine learning because source and target datasets often differ in acquisition protocols, patient demographics, label definitions, and image quality. EchoNet-Dynamic and CAMUS are both public echocardiography resources, but they differ in video format, spatial resolution, frame count, annotation type, released EF labels, and EF distribution [7, 21]. A naive comparison across these datasets can confound performance transfer with target-definition mismatch. For this reason, preprocessing and EF harmonization are not merely technical details; they define the statistical meaning of the evaluation. In this study, CAMUS is converted to an EchoNet-compatible representation and its EF target is recalculated with an apical-four-chamber monoplane approximation. This target is used as an explicitly defined external-cohort endpoint, while acknowledging that the exact clinical method used to generate the released EchoNet-Dynamic EF labels is not specified in the public documentation.

## Materials and methods

### Weakly supervised EF formulation

Let $X_i=\{x_{i,t}\}_{t=1}^{T_i}$ denote an echocardiography video and let $y_i\in\mathbb{R}$ be the corresponding EF value. The weakly supervised EF problem is to estimate $y_i$ from $X_i$ without requiring a dense annotation for every frame. In the strictest form, the training set contains only pairs $(X_i,y_i).$ In the setting studied here, a limited set of frame-level contours is available for learning an auxiliary anatomical representation, but the final regression objective remains video-level:

1 $$\mathcal{L}_{EF}(\theta)=\frac{1}{N}\sum_{i=1}^{N}\ell\left(f_{\theta}(X_i),y_i\right),$$

where $f_{\theta}$ denotes the EF estimator and *ℓ* is an absolute or squared regression loss. When segmentation annotations $G_{i,t}$ exist for a subset of frames, an auxiliary segmentation loss can be used to learn an anatomical encoder or mask predictor:

2 $$\mathcal{L}_{seg}(\phi)=\frac{1}{|\Omega|}\sum_{(i,t)\in\Omega}\ell_{seg}\left(s_{\phi}(x_{i,t}),G_{i,t}\right),$$

with *Ω* denoting the annotated frame subset. The EF model can then operate on masks or mask-derived features rather than raw frames. This decomposition makes the annotation assumptions explicit: dense labels improve the anatomical bottleneck but are not required as labels for the EF regressor. Table 1 summarizes these annotation assumptions.

**Table 1:** Annotation assumptions of the main model families considered in this study

Model family	Training supervision	Inference input	Annotation-efficiency interpretation
Direct video regression	Video-level EF labels	Echocardiography video	Weak supervision, but no anatomical constraint
Segmentation-based EF	Frame contours or masks plus EF labels	Video and predicted masks	Dense labels needed for segmentation, interpretable geometry
Contrastive pretraining	Unlabeled frames/videos plus limited masks	Video	Reduces dependence on dense labels for representation learning
Feature-based CAFEx	Limited masks for anatomical bottleneck plus video-level EF	Video only	Weak EF supervision with compact anatomical representation

The table distinguishes between labels needed during training and information required at inference

This formulation is relevant for studies that aim to use routinely available clinical data. Video-level EF labels are generated routinely and can be collected retrospectively at scale, whereas dense contours typically require additional expert effort. A pipeline that uses sparse dense annotations to learn a stable representation and then relies on weak video-level labels for EF regression is therefore more scalable than a fully dense annotation protocol. At the same time, it is less unconstrained than pure direct regression, because the anatomical bottleneck discourages predictions based solely on non-causal appearance correlations.

### Datasets

Two public echocardiography datasets are used. EchoNet-Dynamic contains 10,030 apical four-chamber echocardiography videos acquired at Stanford University between 2016 and 2018 [7, 20, 31]. Each video is stored in AVI format at *112 × 112* pixels and contains 28 to 1002 frames. The released files provide clinical EF, ED volume (EDV), and ES volume (ESV) labels together with sparse ED and ES left-ventricular tracings. The public dataset description and original EchoNet-Dynamic article do not explicitly state that the released EF labels were generated with a monoplane Simpson method; therefore, we treat the EchoNet-Dynamic EF values as released clinical labels and avoid making a stronger claim about their exact clinical measurement procedure.

**Fig. 1:**
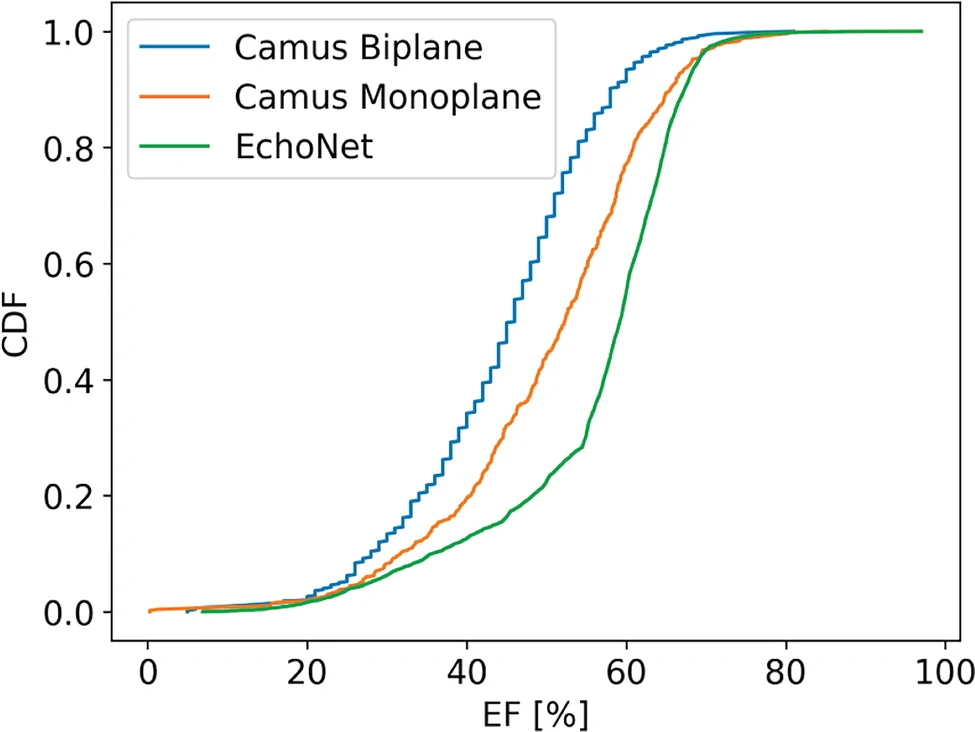
Distribution of EF values across datasets and EF calculation variants. EchoNet-Dynamic and CAMUS differ not only in image format and annotation type, but also in their EF distributions and released target definitions

CAMUS contains 500 patients with apical two- and four-chamber echocardiography sequences stored in NIfTI format [21, 22]. In contrast to EchoNet-Dynamic, CAMUS videos have variable spatial resolution, contain 10 to 42 frames, and include dense pixel-wise labels for all frames. CAMUS also provides image-quality categories (good, medium, and poor) and EF values derived from biplane Simpson calculation, which is the preferred 2D approach when both apical views are available [5]. Table 2 summarizes the most important differences. The corresponding EF distributions are shown in Fig. 1. These differences motivate the preprocessing steps described below.

**Table 2:** Comparison of the EchoNet-Dynamic and CAMUS datasets used in this study

	EchoNet-Dynamic	CAMUS
Videos	10,030	500
Video format	AVI	NIfTI
Resolution	*112 × 112*	*322*–*1180 × 292*–*972*
Frames per video	28–1002	10–42
Total frames	1,770,636	9,964
Annotated frames	20,060	9,964
Segmentation	42 endocardial points	Dense pixel labels
Released EF labels	Clinical EF/EDV/ESV labels; exact public EF calculation method not specified	Biplane Simpson EF
EF minimum [%]	6.91	0.27
EF maximum [%]	96.97	85.08
EF mean [%]	55.75	50.37

### Problem formulation and metrics

For a video $X_i$ with ground-truth EF $y_i$, the EF estimation task is to learn a function $f_\theta$ that predicts $\hat{y}_i=f_\theta(X_i).$ When a model produces a segmentation mask *S*, segmentation accuracy is quantified with the Dice similarity coefficient

3 $$\mathrm{DSC}(S, G) = \frac{2|S \cap G|}{|S| + |G|},$$

where *G* denotes the ground-truth segmentation. EF regression is evaluated with mean absolute error (MAE)

4 $$\mathrm{MAE} = \frac{1}{N}\sum_{i=1}^{N}|y_i-\hat{y}_i|,$$

and coefficient of determination

5 $$R^2 = 1 - \frac{\sum_{i=1}^{N}(y_i-\hat{y}_i)^2}{\sum_{i=1}^{N}(y_i-\bar{y})^2}.$$

Although MAE is clinically interpretable in EF percentage points, $R^2$ is emphasized for cross-dataset comparisons because the EF distributions differ between EchoNet-Dynamic and CAMUS. A model that simply predicts values near the training-set mean can obtain a deceptively moderate MAE while having low or negative explanatory power on a shifted target distribution.

### CAMUS preprocessing

A controlled cross-dataset evaluation requires converting CAMUS into a representation compatible with models trained on EchoNet-Dynamic. CAMUS NIfTI files are converted into video frames, cropped to the cardiac field of view, and resized to the *112 × 112* EchoNet-Dynamic input format. The corresponding dense segmentation labels are transformed with the same operations. CAMUS endocardial masks are then converted into binary LV masks comparable to EchoNet-Dynamic tracings, and EF values are recalculated from the converted apical four-chamber segmentations using a monoplane Simpson approximation. This step defines a single-plane CAMUS endpoint for the external evaluation; it should not be read as evidence that the released EchoNet-Dynamic EF labels were themselves explicitly computed by the same monoplane procedure.

**Fig. 2:**
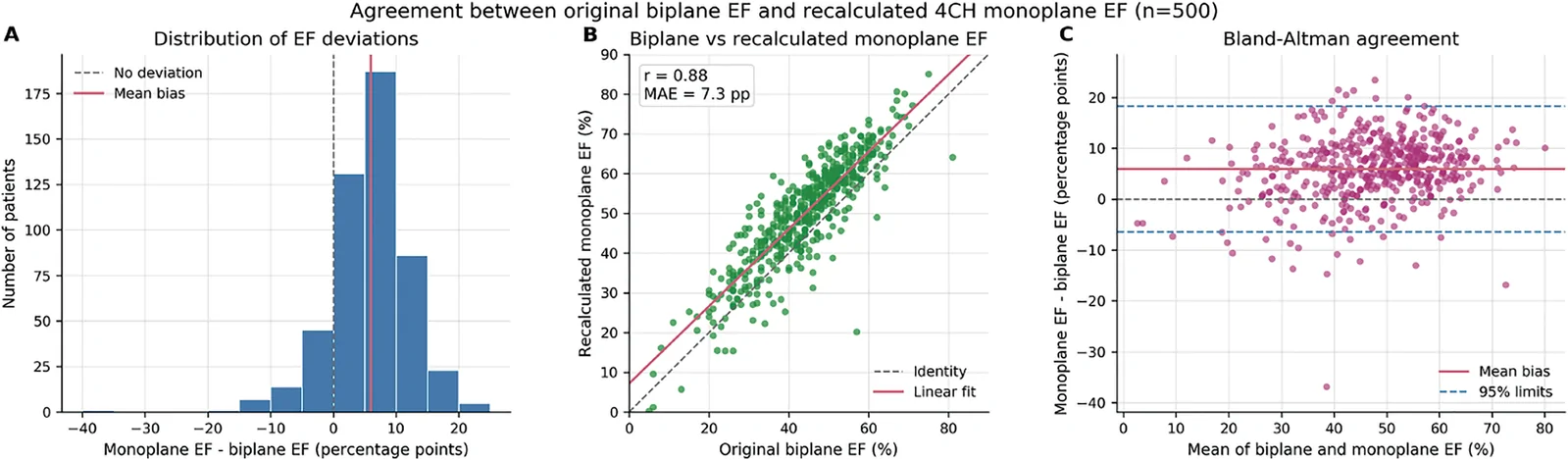
Target harmonization for CAMUS. Panel (**A**) shows the distribution of EF deviations between the recalculated apical-four-chamber monoplane endpoint and the original CAMUS biplane EF, with the zero-deviation reference and mean bias indicated. Panel (**B**) compares original biplane EF with recalculated monoplane EF and reports the correlation and mean absolute error between the two endpoints. Panel (**C**) shows the bland–Altman agreement plot, with mean bias and 95% limits of agreement. The recalculated monoplane endpoint is used as the explicit single-plane target for the external evaluation, but the observed bias and limits of agreement indicate that it should not be treated as identical to the original CAMUS biplane reference

The choice to report a recalculated CAMUS EF endpoint is important. Without harmonization, the experiment would compare models trained on released EchoNet-Dynamic clinical EF labels against original CAMUS biplane labels, confounding dataset shift with target-definition shift. The harmonized protocol does not remove all differences between datasets, and it does not make the CAMUS target identical to EchoNet-Dynamic. It does, however, make the external evaluation more explicit by using an apical-four-chamber endpoint derived from the same view used by the input videos and tracings. The target-harmonization analysis in Fig. 2 shows that the recalculated four-chamber monoplane EF is strongly correlated with the original CAMUS biplane EF (*r=0.88*), but also has a non-negligible absolute deviation (MAE = 7.3 percentage points), positive mean bias, and visible limits of agreement. This supports using the recalculated value as a transparent single-plane external endpoint, while avoiding the claim that it is interchangeable with the original biplane clinical reference.

This harmonization step also clarifies what the external-cohort experiment measures. The evaluation is not designed to test whether a model trained on EchoNet-Dynamic can reproduce the original CAMUS biplane labels. Instead, it asks whether models trained on EchoNet-Dynamic can retain segmentation and EF-prediction performance when the image distribution, annotation density, frame count, and acquisition quality differ, while the external EF endpoint is defined from the apical four-chamber view in a transparent and reproducible way.

### Baseline models

The benchmark includes models representative of the main methodological families in automated EF estimation. EchoNet-Dynamic is used as a segmentation-based reference model with DeepLabV3-ResNet50 for left ventricular segmentation and EF estimation [7]. This baseline is the conventional ED/ES segmentation-to-volume comparison in the benchmark: the model predicts the LV endocardial cavity on ED and ES frames, and EF is then derived from the predicted ED and ES geometry rather than from a direct video-regression head. EchoCoTr represents direct transformer-based video regression using a UniFormer-style architecture [11]. EchoGNN represents graph-based temporal reasoning over learned frame embeddings [14]. The feature-extraction baseline reproduces the anatomical approach of Batool et al., which predicts EF from segmentation-derived short-axis features [15]. EchoConPre evaluates the effect of contrastive pretraining on segmentation [18]. EchoSwin evaluates transformer-based segmentation [32]. An augmentation-based segmentation model inspired by GUDU is included to analyze whether echocardiography-specific augmentation is associated with smaller external-cohort degradation [33].

The goal is not to claim a definitive reimplementation of every baseline, but to compare the models under a consistent train-on-EchoNet/test-on-CAMUS protocol. Hyperparameters follow the corresponding publications whenever possible, with practical adjustments for memory and convergence. Table 3 reports the reproduction check on EchoNet-Dynamic.

**Table 3:** Reproduction check on EchoNet-Dynamic

Model	DSC [%]		MAE [%]	
	Paper	This study	Paper	This study
EchoNet-Dynamic [7]	92.00	91.81	4.05	9.09
EchoCoTr [11]	–	–	3.95	4.00
EchoConPre [18]	92.52	92.14	–	–
EchoGNN [14]	–	–	4.45	4.45
EchoSwin [32]	92.92	90.00	–	–
Feature-extraction baseline [15]	92.05	90.74	5.74	5.78

Dice score and MAE are reported when applicable

### CAFEx architecture

CAFEx is designed around the hypothesis that external-cohort EF estimation may benefit from an explicit anatomical bottleneck. Instead of directly regressing EF from raw pixels, CAFEx first predicts the left ventricular segmentation, then extracts temporal features from the mask sequence, and finally predicts EF from these features. The acronym denotes the main components: contrastive pretraining (C), echocardiography-specific augmentation (A), feature extraction (F), and EF regression from extracted features (Ex).

The segmentation component is based on DeepLabV3 with a ResNet50 backbone. Contrastive pretraining with SimCLR-style augmentations is used to improve representation quality before supervised fine-tuning. During segmentation fine-tuning, specialized data augmentation is applied to mimic plausible echocardiographic variation, including intensity and contrast changes, geometric perturbations, and image-quality degradation. This combination aims to make the segmentation model less dependent on EchoNet-specific appearance.

**Fig. 3:**
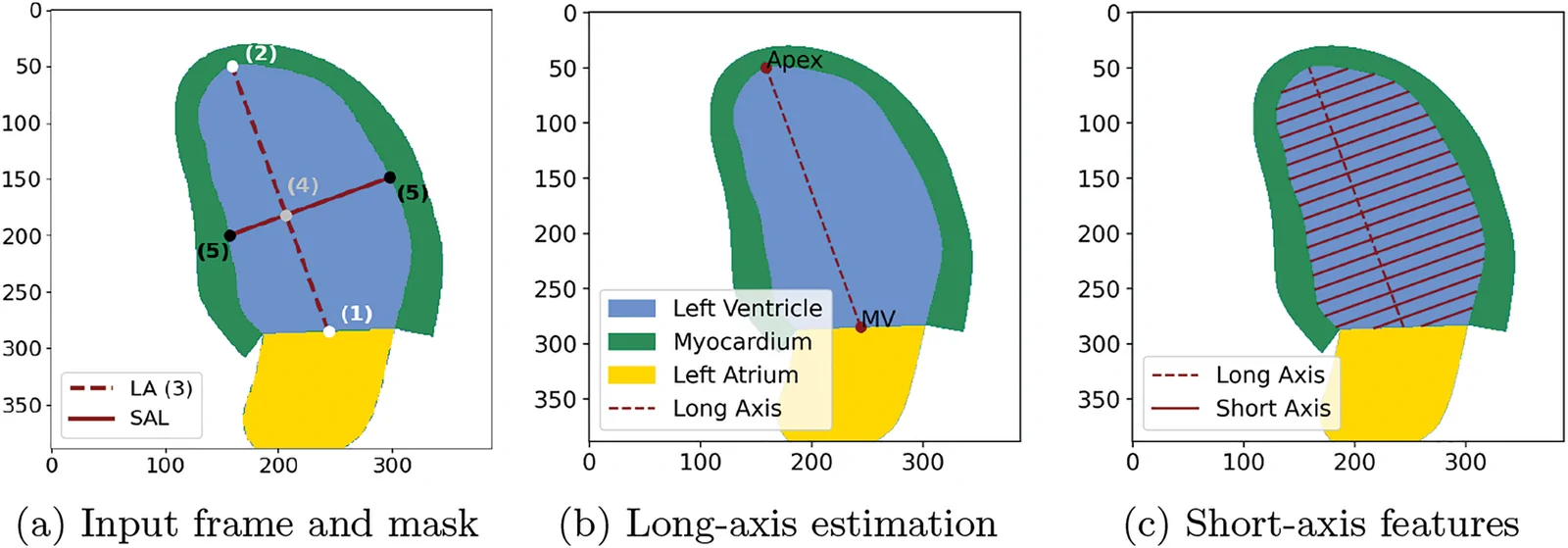
Geometric feature extraction from a predicted left ventricular segmentation. The line-length feature family approximates the clinical Simpson workflow by estimating the long axis and measuring short-axis diameters along the ventricle

From the predicted segmentation masks, CAFEx extracts two alternative feature families. The first family follows clinical geometry and uses the lengths of short-axis lines derived from the endocardial contour. The second uses the number of pixels classified as left ventricle as a simpler area-based descriptor. Both are evaluated because geometric line extraction requires assumptions about the apex and mitral valve plane, whereas area features are less structured but potentially less sensitive to noisy contour geometry. Figure 3 illustrates the geometric feature extraction.

### Mask-derived feature extraction

Feature extraction is deterministic once the segmentation mask sequence has been predicted. For each sampled frame, the LV probability map is binarized and the largest connected LV component is retained. Small holes are filled before contour extraction so that the subsequent measurements are based on one closed endocardial region. For the line-length feature family, the endocardial contour is used to estimate a long axis from the basal region toward the apex. Short-axis chords are then placed approximately perpendicular to this long axis at fixed relative positions between base and apex. The resulting feature vector for frame *t* is

6 $$\mathbf{l}_{t} = \left[l_{t,1}, l_{t,2}, \ldots, l_{t,n}\right],$$

where $l_{t,k}$ denotes the length of the *k*-th short-axis chord through the predicted LV mask. The same number and relative positions of chords are used for every frame and video, producing a fixed-dimensional anatomical descriptor. Lengths are measured in the resized *112 × 112* image space and normalized before temporal regression to reduce dependence on absolute image scale.

The area feature family uses the same binarized LV masks but avoids apex and basal-plane estimation. For each frame, the descriptor is the normalized LV area

7 $$a_t = \frac{|S_t|}{112 \times 112},$$

where $S_t$ is the predicted LV mask. Thus, the EF regressor receives either a sequence of short-axis length vectors $\{\mathbf{l}_t\}$ or a sequence of normalized areas $\{a_t\}$. In the ED/ES frame mode, only the selected ED and ES descriptors are passed to the temporal model. In the video mode, descriptors are extracted from the sampled frame sequence described below. Variable-length sequences are padded by appending the feature sequence in reverse order, which preserves plausible temporal continuity better than zero padding in this setting. The final LSTM or BERT-style regressor maps this fixed-size feature sequence to a single video-level EF estimate.

Temporal regression is performed with either a long short-term memory (LSTM) model or a BERT-style transformer. Two sampling modes are considered. The frame-based mode uses the annotated ED and ES frames, representing the setting where phase information is known. The video-based mode samples every second frame for short videos and every third frame from the first 100 frames for longer videos. Since videos have variable length, sequences are padded. Instead of appending zeros, which degraded performance, CAFEx pads by appending the feature sequence in reverse order, approximating the continuation of a cardiac cycle and avoiding abrupt non-physiological zero features.

**Fig. 4:**
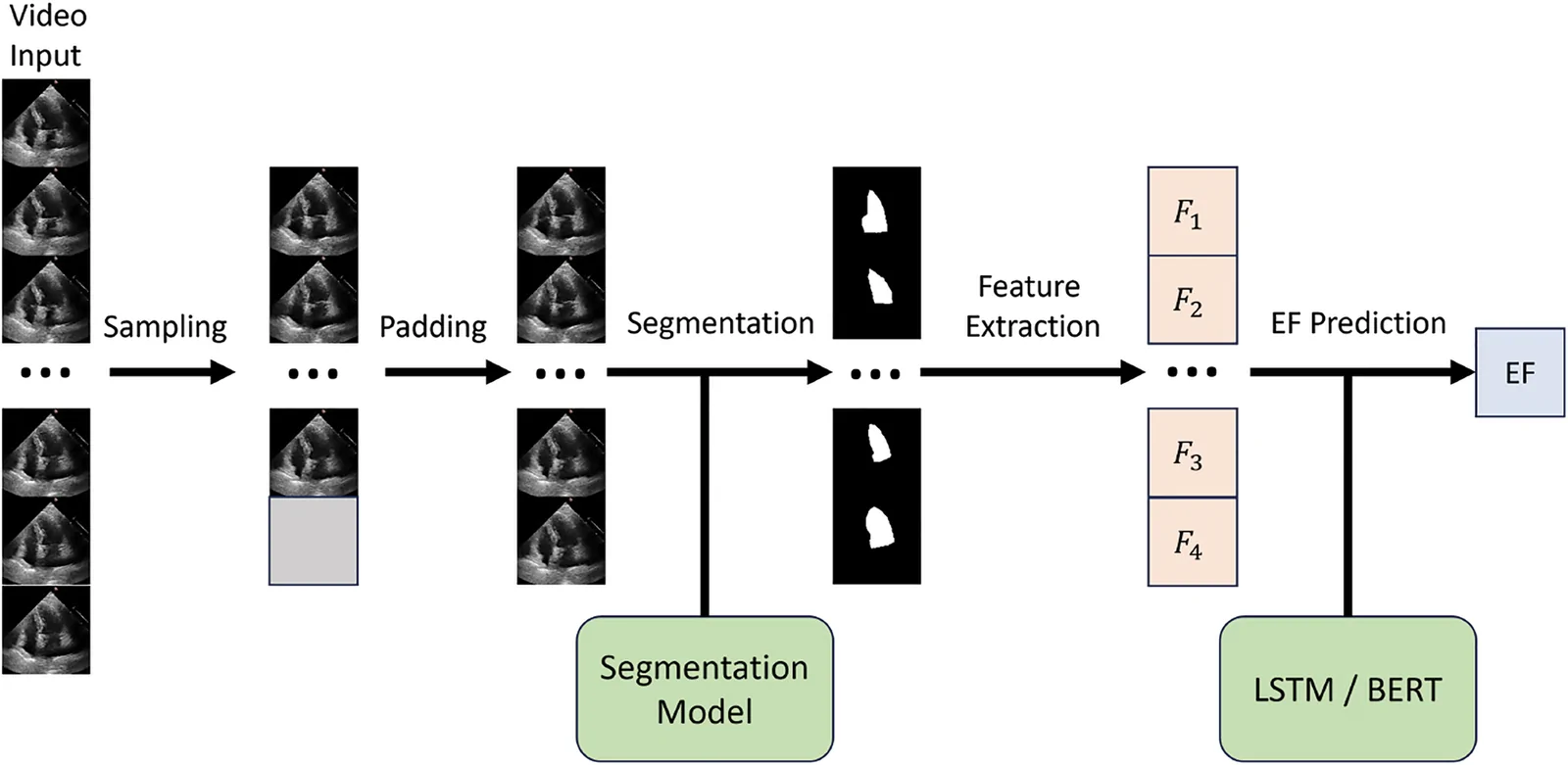
CAFEx prediction pipeline. The input video is sampled and padded, a contrastive-augmented segmentation model predicts left ventricular masks, anatomical features $F_n$ are extracted from each mask, and a temporal regressor predicts EF

The explicit feature representation is intended to limit the hypothesis space of the EF regressor. A direct video model must learn image appearance, cardiac phase, ventricular geometry, and target calibration simultaneously. CAFEx separates these components: the segmentation network localizes the chamber, the feature extractor converts the mask into compact anatomical measurements, and the temporal regressor maps the cardiac-cycle feature sequence to EF. This decomposition can discard appearance variation that is irrelevant for EF and preserve the measurements most closely related to volume change. Its main weakness is that errors in basal contour extraction, apex localization, or ED/ES phase representation can propagate to the final estimate. The complete CAFEx pipeline is shown in Fig. 4.

### Annotation-efficient role of the anatomical bottleneck

The anatomical bottleneck in CAFEx is deliberately low-dimensional. It converts a high-dimensional ultrasound video into a sequence of ventricular measurements or areas. This design has two consequences. First, it removes much of the acquisition-specific image appearance before EF regression. Speckle statistics, gain, sector shape, and background structures may influence segmentation, but they are not directly available to the final regressor. Second, it constrains the EF estimate to depend on quantities that are mechanically related to ventricular emptying. The bottleneck therefore acts as a weak form of inductive supervision: it does not provide the EF label itself, but it restricts the representation through which the EF label can be predicted.

**Fig. 5:**
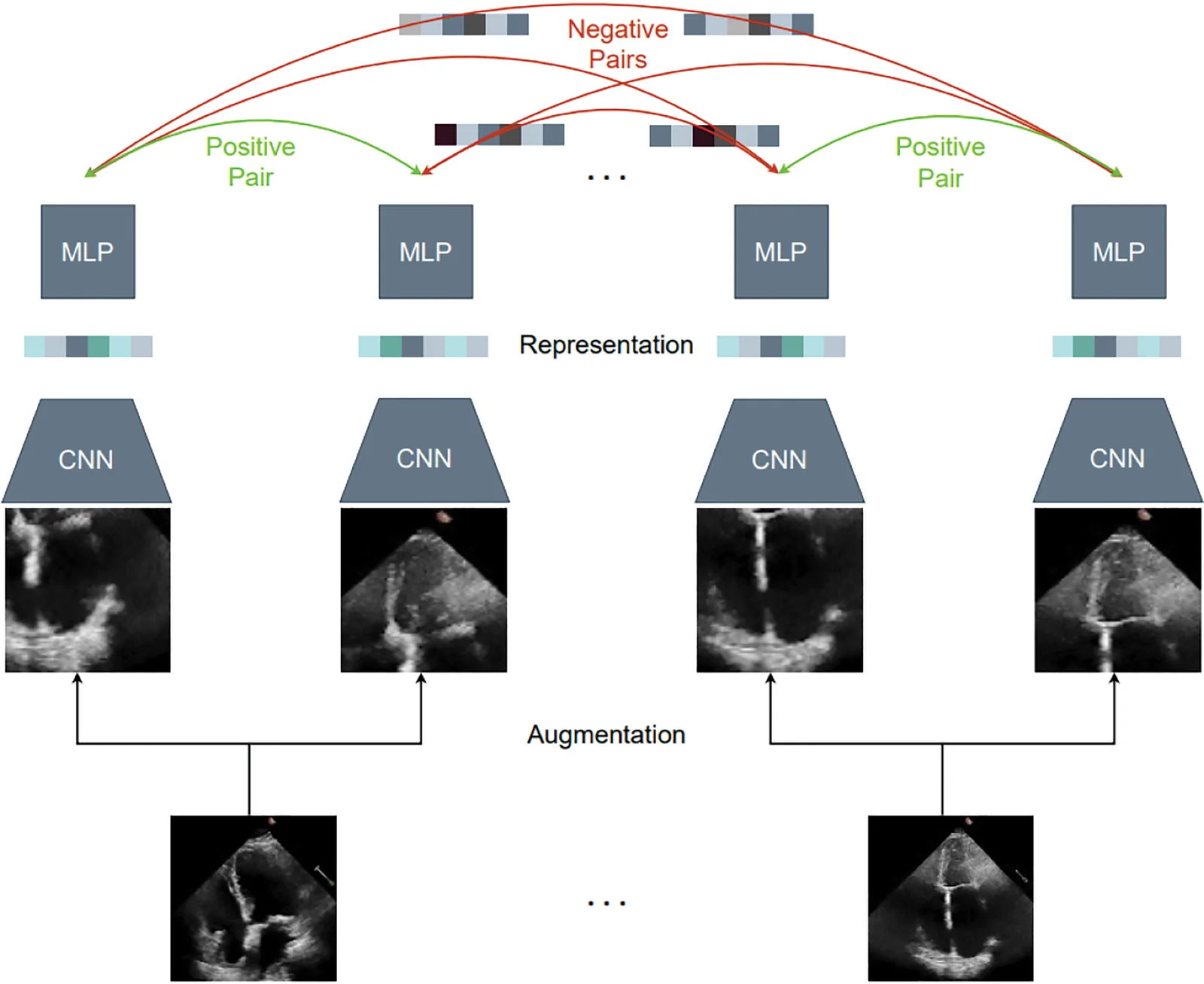
Contrastive pretraining used for the segmentation backbone. Two augmented views of the same echocardiographic frame are encouraged to have nearby representations, whereas different frames are pushed apart. This stage uses image structure rather than manual EF or mask labels and is therefore compatible with annotation-efficient training

This is distinct from simply adding a segmentation module for interpretability. If segmentation is used only to visualize a direct model, the EF regressor may still rely on non-anatomical features. In CAFEx, the regressor receives only extracted features, and thus the anatomical representation is part of the computational path. Such a design is particularly useful in low-annotation scenarios because it enables the dense labels from a small annotated subset to regularize the learning problem for a much larger set of weakly labeled videos. The contrastive pretraining step is illustrated in Fig. 5.

### Data harmonization as part of the evaluation protocol

The train-on-EchoNet/test-on-CAMUS evaluation requires harmonization at three levels. The first is image harmonization. CAMUS frames have variable resolution and are stored in NIfTI format, whereas EchoNet-Dynamic uses fixed-size video frames. Resizing CAMUS to the EchoNet input size enables the same trained networks to be applied without architectural changes, but it also reduces spatial detail. The second is annotation harmonization. EchoNet-Dynamic provides sparse contour points, while CAMUS provides dense labels. For a fair segmentation comparison, both are converted into binary left-ventricular masks in the resized image space. The third is target harmonization. EchoNet-Dynamic provides released clinical EF labels and apical-four-chamber tracings, while CAMUS provides biplane EF values. We therefore recalculate CAMUS EF from the apical-four-chamber segmentation to define an explicit single-plane external endpoint, while recognizing that this approximation is not guaranteed to match the undocumented clinical procedure used for the released EchoNet-Dynamic EF labels.

This protocol intentionally does not remove all dataset differences. Image texture, scanner characteristics, frame count, field of view, population, and quality distribution remain different. The objective is not to make CAMUS indistinguishable from EchoNet-Dynamic, but to remove avoidable inconsistencies that would otherwise make the external evaluation uninterpretable. After harmonization, a remaining performance gap can more plausibly be attributed to the evaluated distribution shift and representation sensitivity.

### Training details

All models are trained on EchoNet-Dynamic training data and evaluated on the EchoNet-Dynamic test set and the preprocessed CAMUS set without CAMUS fine-tuning. This design isolates performance on the harmonized external cohort. For models requiring segmentation supervision, EchoNet-Dynamic ED/ES tracings are converted to binary LV cavity masks. CAMUS masks are used only for evaluation. Table 4 lists the main training settings. No exhaustive hyperparameter search is performed; the experiments are intended to evaluate performance-transfer trends and the effect of architectural choices under a consistent protocol.

**Table 4:** Main hyperparameters used for the evaluated models

Model	GPU	Batch	Epochs	Learning rate	Optimizer / pretraining
EchoNet-Dynamic [7]	RTX 2080 Ti	20	50	$10^{-5}$	SGD / ResNet50
EchoCoTr [11]	RTX 4090	25	45	$10^{-4}$	AdamW / UniFormer K400
Contrastive pretraining [18]	RTX 2080 Ti	128	100	$10^{-3}$	Adam / ResNet50
EchoConPre [18]	RTX 4090	16	50	$10^{-4}$	MADGRAD / contrastive
EchoGNN [14]	RTX 4090	80	10,000	$10^{-4}$	Adam / frame attention
Feature baseline [15]	RTX 2060	16	50	$10^{-4}$	SGD / ResNet50
EchoSwin [32]	RTX 2060	32	100	$10^{-3}$	Adam / none
Augmentation model [33]	RTX 2080 Ti	32	20	$10^{-3}$	SGD / ResNet50
CAFEx (ours)	RTX 2060	16	100	$10^{-4}$	Adam / ResNet50

Citations indicate the source method or methodological component when applicable

### Computational considerations

CAFEx is a multi-stage pipeline and is therefore computationally less direct than a single video-to-EF regressor. At inference, the sampled frames are first processed by the segmentation backbone, then converted into low-dimensional anatomical descriptors, and finally passed to the temporal regressor. The feature-extraction and LSTM stages are lightweight compared with the DeepLabV3-ResNet50 segmentation backbone, but the need to segment sampled frames introduces an overhead that direct video-regression approaches do not have. Conversely, direct video models process raw image sequences with larger temporal backbones and can have higher memory requirements depending on the number of frames and attention operations.

The experiments in this study were designed to compare external-cohort accuracy under a common train-on-EchoNet/test-on-CAMUS protocol rather than to provide a deployment benchmark. Standardized per-video inference times, FLOPs, and parameter counts were not recorded for all reproduced baselines under identical software and hardware conditions. Consequently, the reported CAFEx gains should be interpreted as accuracy and robustness gains in this benchmark, not as evidence of superior computational efficiency. A deployment-oriented comparison should measure inference latency, memory use, parameter count, and throughput for each method under the same frame-sampling policy and hardware.

## Results

### Cross-dataset benchmark

Table 5 summarizes the main cross-dataset benchmark. All models were trained or selected according to the EchoNet-Dynamic setting and were subsequently evaluated on the harmonized CAMUS cohort. The first observation is that in-distribution performance on EchoNet-Dynamic is a weak proxy for external performance. Direct EF regressors obtain high results on the source dataset, but their behavior changes markedly on CAMUS. EchoCoTr achieves the highest in-distribution $R^2$ score among the reproduced EF models $(R^2=0.81),$ but decreases to $R^2=-0.43$ on CAMUS. EchoGNN follows the same pattern, decreasing from $R^2=0.76$ to $R^2=-0.80$. These negative values indicate that, on the external cohort, the models explain less variance than a constant predictor based on the target mean.

**Table 5:** Cross-dataset performance when models are trained on EchoNet-Dynamic and tested on EchoNet-Dynamic and CAMUS

Model	DSC [%]			$R^2$		
	EchoNet	CAMUS	Difference	EchoNet	CAMUS	Difference
EchoNet-Dynamic [7]	91.81	89.06	−2.72	0.80	0.28	−0.52
EchoCoTr [11]	–	–	–	**0.81**	−0.43	−1.24
EchoConPre [18]	92.14	**91.08**	**−1.06**	–	–	–
EchoGNN [14]	–	–	–	0.76	−0.80	−1.56
EchoSwin [32]	90.00	84.78	−5.22	–	–	–
EchoSwin + augmentation [32, 33]	92.39	89.53	−2.86	–	–	–
Feature baseline [15]	90.74	87.41	−3.33	0.60	0.38	−0.22
Augmentation model [33]	91.85	89.87	−1.71	–	–	–
**CAFEx**	92.10	90.73	−1.37	0.69	**0.54**	**−0.15**
SimLVSeg [19]	**93.32**	90.62	−2.70	–	–	–

Dice scores are reported for binary left-ventricular cavity masks on ED/ES frames. The EchoNet-Dynamic row is the conventional ED/ES segmentation-to-volume baseline from Ouyang et al.; its CAMUS Dice score is obtained by direct evaluation on harmonized CAMUS masks without CAMUS fine-tuning. SimLVSeg is included as an external literature result and was not reproduced in this study

Feature-based models show a different pattern. The reproduced feature-extraction baseline obtains a lower source-domain $R^2$ than the strongest direct video regressor, but retains positive explanatory power on CAMUS. CAFEx further improves this external-benchmark result, increasing CAMUS $R^2$ from 0.38 to 0.54 while reducing the CAMUS MAE from 7.89% to 6.70%. This finding is central to the study: the anatomically constrained representation is not merely a post-hoc interpretation layer, but is associated with different external-benchmark behavior of the EF predictor. The source-domain result remains competitive ($R^2=0.69$), while the loss in performance under the evaluated dataset shift is smaller than for the direct-regression baselines.

For segmentation, the external degradation is present but less pronounced than for EF regression. The EchoNet-Dynamic segmentation baseline decreases from 91.81% Dice on EchoNet-Dynamic to 89.06% on CAMUS. This row reports direct evaluation of the EchoNet-Dynamic-trained LV cavity segmentation model on the harmonized CAMUS masks, without CAMUS fine-tuning; the Dice score is calculated only for the left-ventricular cavity. EchoSwin without augmentation decreases from 90.00% to 84.78%, whereas the augmentation-enhanced version improves the CAMUS Dice score to 89.53%. Contrastive pretraining yields the strongest reproduced CAMUS segmentation Dice among the segmentation baselines (91.08%). CAFEx obtains 90.73% Dice after integrating contrastive pretraining into the feature-extraction pipeline. Thus, the segmentation results show that anatomical localization shows smaller degradation than direct EF regression in this benchmark, although mask quality remains an important source of downstream error.

**Fig. 6:**
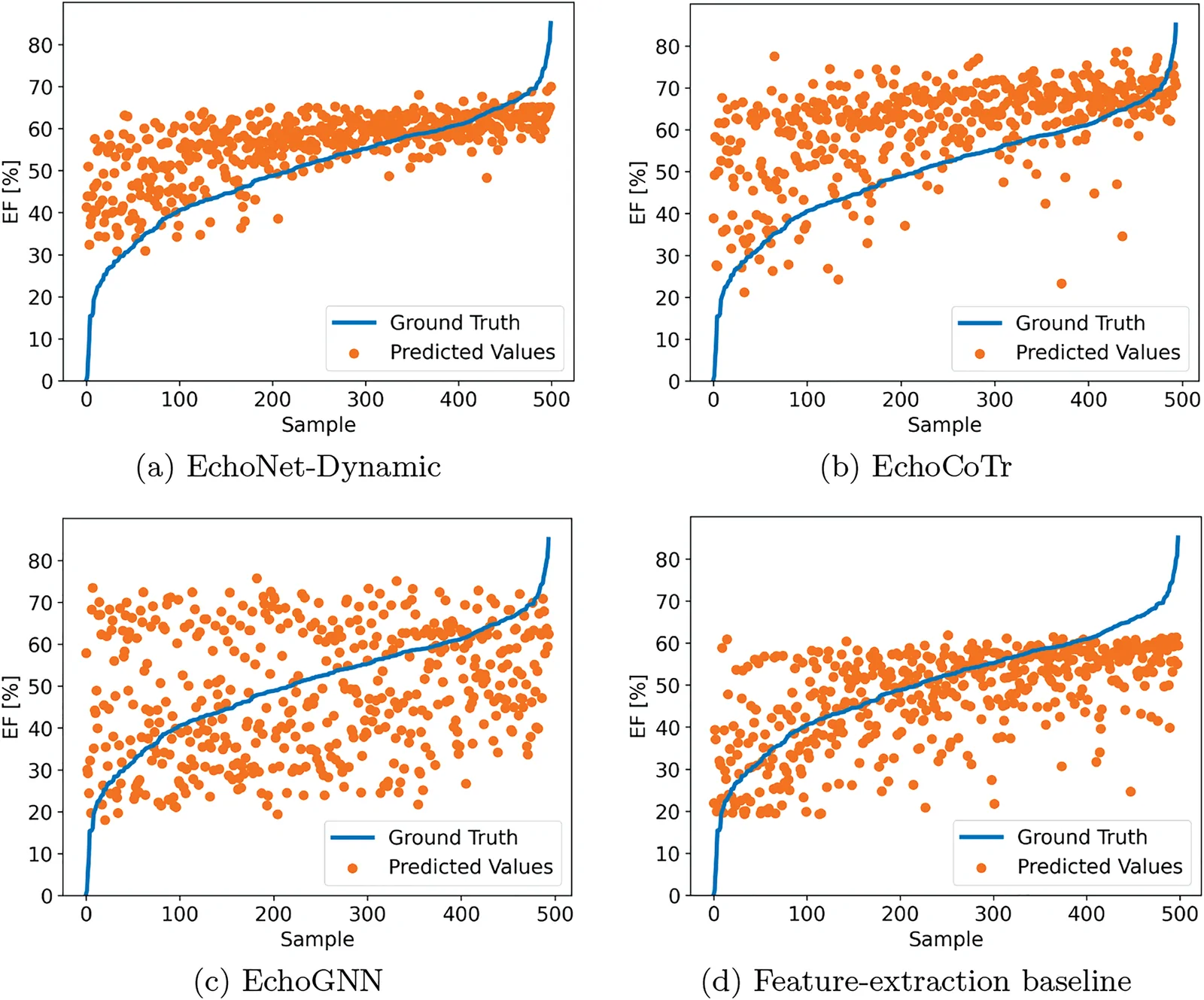
CAMUS EF predictions from representative baselines. In each panel, samples are ordered by ground-truth EF; the blue curve represents sorted ground-truth EF values, and orange markers represent the corresponding predictions at the same sorted indices. These plots are intended as qualitative error-structure visualizations rather than substitutes for agreement plots. Direct video-regression approaches show substantial error structure in the evaluated dataset shift, whereas feature-based prediction is less degraded in this benchmark

**Fig. 7:**
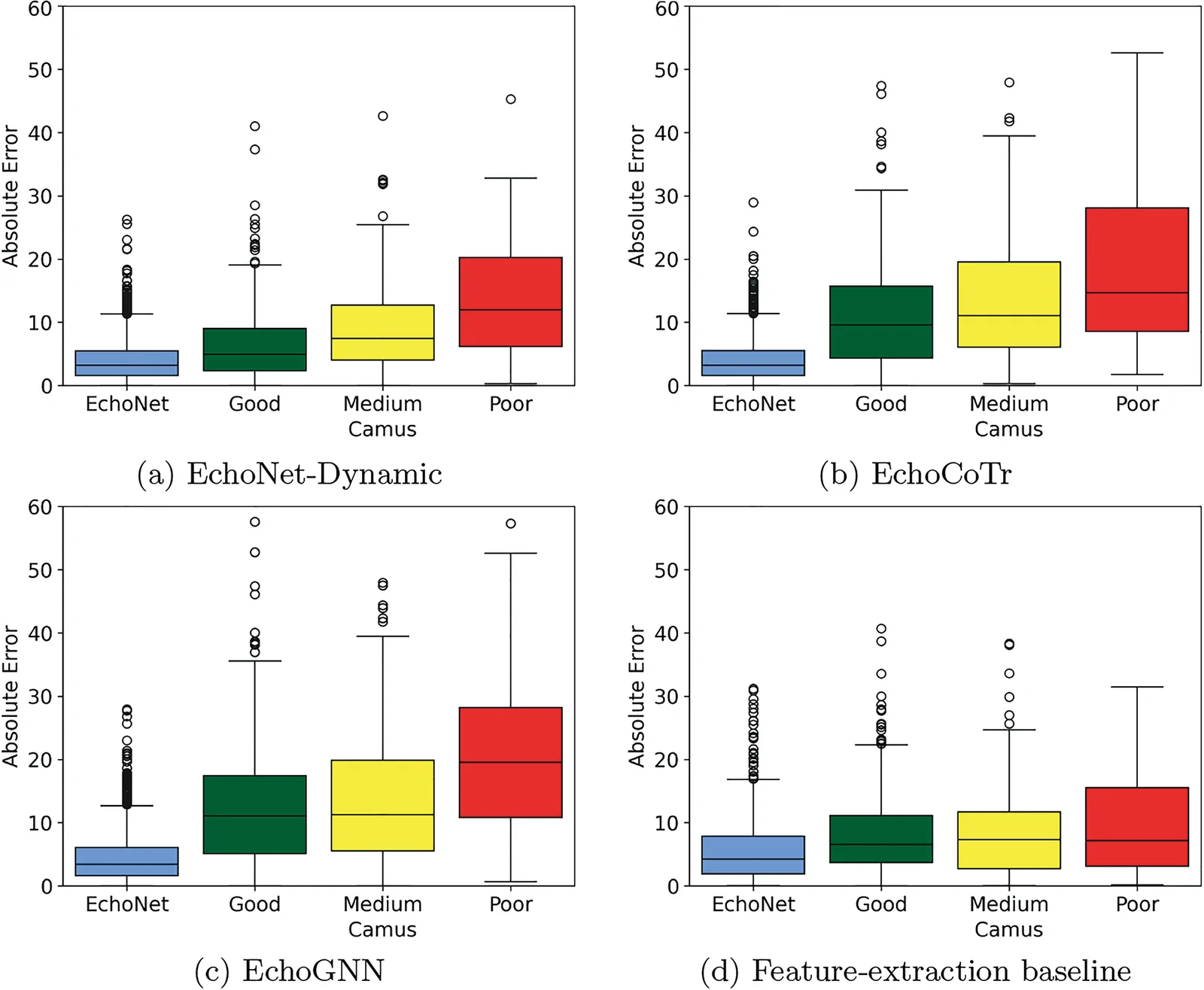
Absolute EF prediction errors of representative baselines stratified by dataset and CAMUS video quality. The x-axis categories are the EchoNet-Dynamic test set and the CAMUS good-, medium-, and poor-quality subsets. The quality stratification reveals that poor-quality videos are challenging in this benchmark, but model architecture is associated with the magnitude of external-benchmark degradation

The prediction curves in Fig. 6 show that the negative $R^2$ values of direct models are not caused only by isolated outliers. They reflect systematic mismatch between the learned prediction range and the external target distribution. High EF values are often underestimated, whereas low EF values show high variance. The quality-stratified errors in Fig. 7 further show that low image quality amplifies this error pattern, but even good-quality CAMUS videos can remain difficult if the model relies on dataset-specific appearance rather than on ventricular anatomy. These boxplots report the distribution of absolute errors, including median, interquartile range, whiskers, and outliers, and therefore complement the mean MAE and $R^2$ values in the tables.

### Component analysis of CAFEx

The CAFEx design was analyzed by varying the segmentation model, temporal regressor, frame sampling strategy, and feature representation. Table 6 reports the resulting combinations. The first row, DLV3–LSTM–F–L, corresponds to the reproduced Batool et al. feature-extraction baseline and serves as the direct comparison for the newly introduced CAFEx components. The notation is as follows: DLV3 denotes the DeepLabV3 baseline segmentation model; Aug denotes DeepLabV3 with data augmentation; CPAug denotes contrastive pretraining followed by augmentation fine-tuning; LSTM and BERT denote the EF regressors; F denotes ED/ES frame sampling; V denotes video sampling; L denotes line-length features; and A denotes segmentation-area features.

**Table 6:** CAFEx ablation over segmentation model, temporal regressor, frame sampling, and feature type

Model combination	MAE [%]		$R^2$	
	EchoNet	CAMUS	EchoNet	CAMUS
DLV3–LSTM–F–L [15]	5.78	7.89	0.60	0.38
Aug–LSTM–F–L	5.51	7.23	0.61	0.48
Aug–LSTM–F–A	5.43	7.15	0.62	0.50
Aug–LSTM–V–L	4.97	8.06	0.69	0.35
Aug–LSTM–V–A	5.25	10.38	0.65	0.09
Aug–BERT–F–L	5.54	7.69	0.62	0.43
Aug–BERT–F–A	5.88	7.56	0.57	0.44
Aug–BERT–V–L	4.98	8.62	0.68	0.30
Aug–BERT–V–A	5.20	8.17	0.65	0.37
CPAug–LSTM–F–L	4.88	6.70	0.69	**0.54**
CPAug–LSTM–F–A	5.08	**6.69**	0.66	**0.54**
CPAug–LSTM–V–L	4.77	7.33	0.72	0.45
CPAug–LSTM–V–A	5.04	10.43	0.67	−0.12
CPAug–BERT–F–L	5.01	7.25	0.68	0.48
CPAug–BERT–F–A	5.19	6.72	0.64	0.53
CPAug–BERT–V–L	**4.65**	7.72	**0.73**	0.42
CPAug–BERT–V–A	4.91	7.25	0.69	0.48

Several consistent trends emerge from the ablation. First, adding contrastive pretraining to the augmentation-based segmentation model improves most EF configurations. This suggests that the quality of the anatomical masks matters not only for the segmentation metric itself, but also for the stability of the derived EF features. Second, LSTM regression is stronger than the BERT-style transformer when the input is restricted to ED and ES frames. Third, using a longer sampled video improves EchoNet-Dynamic performance in several configurations but tends to hurt CAMUS generalization. This is consistent with the dataset structure: CAMUS videos are shorter, contain only a partial cardiac cycle, and differ in temporal sampling. A temporal representation that is helpful on the source dataset may therefore become a source of mismatch on the external cohort. Fourth, neither line-length nor area features dominate universally. The two best CAMUS configurations are CPAug–LSTM–F–L and CPAug–LSTM–F–A, both reaching $R^2=0.54$.

The standalone segmentation variants are summarized in Table 7. Contrastive pretraining gives the best reproduced CAMUS Dice score. The integrated CAFEx model uses a contrastive-initialized model that is subsequently fine-tuned with augmentation; its Dice score is slightly lower than the best standalone contrastive model but remains above 90% on CAMUS. This implementation compromise was necessary to integrate the segmentation backbone into the downstream feature-extraction pipeline, and it still improves EF prediction substantially over the reproduced Batool et al. feature-extraction configuration.

**Table 7:** Segmentation Dice scores of the segmentation variants used in the feature extraction pipeline

Segmentation model	EchoNet [%]	CAMUS [%]
DeepLabV3 baseline	91.81	89.06
DeepLabV3 + augmentation	91.85	89.87
DeepLabV3 + contrastive pretraining	92.14	91.08
DeepLabV3 + contrastive pretraining + augmentation	92.09	90.73

### External comparison with the feature-extraction baseline

The Batool et al. feature-extraction baseline is the strongest reproduced EF baseline on CAMUS and therefore provides the most direct comparison for CAFEx. Table 8 shows that CAFEx improves both MAE and $R^2$ on EchoNet-Dynamic and CAMUS. On CAMUS, the MAE decreases from 7.89% to 6.70%, and the $R^2$ score increases from 0.38 to 0.54. The improvement is largest for good-quality CAMUS videos, where $R^2$ increases from 0.32 to 0.56 and MAE decreases by 1.57 percentage points. Medium-quality videos also improve. Poor-quality videos remain difficult: both models obtain negative $R^2$, indicating that predictions in this subgroup explain less variance than a mean predictor.

**Table 8:** Comparison between the reproduced Batool et al. feature-extraction baseline and CAFEx stratified by CAMUS video quality and EchoNet-Dynamic

Dataset	Video quality	MAE [%]			$R^2$		
		Feature baseline	CAFEx	Difference	Feature baseline	CAFEx	Difference
CAMUS	Good	7.66	6.09	−1.57	0.32	0.56	0.24
CAMUS	Medium	7.51	6.75	−0.40	0.50	0.60	0.10
CAMUS	Poor	10.58	10.26	−0.32	−0.16	−0.14	0.02
CAMUS	All	7.89	6.70	−1.19	0.38	0.54	0.16
EchoNet	All	5.78	4.88	−0.90	0.60	0.69	0.09

Negative MAE differences indicate improvement

**Fig. 8:**
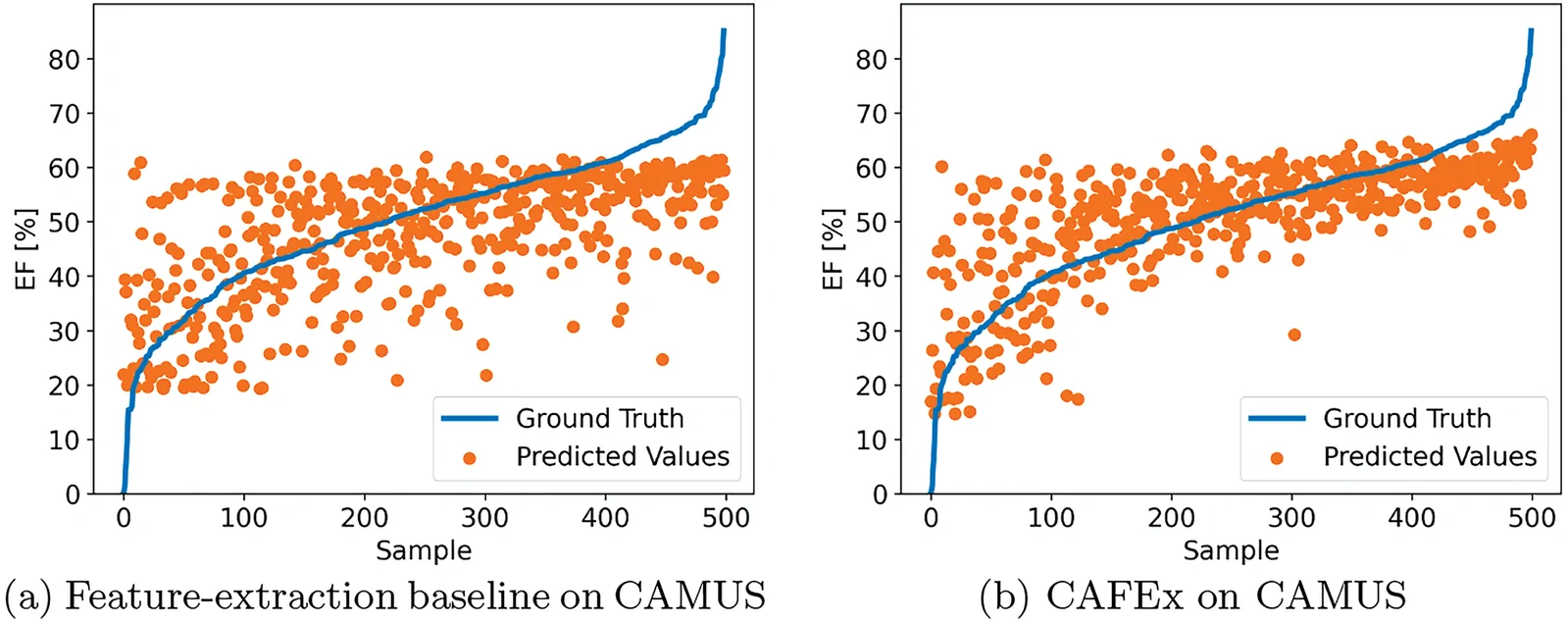
Ground-truth EF values and corresponding predictions on CAMUS, sorted by ground-truth EF. In each panel, the blue curve represents sorted ground-truth EF values, and orange markers represent the corresponding predictions. CAFEx reduces the variance of the prediction errors compared with the feature-extraction baseline, although high EF values remain underestimated

**Fig. 9:**
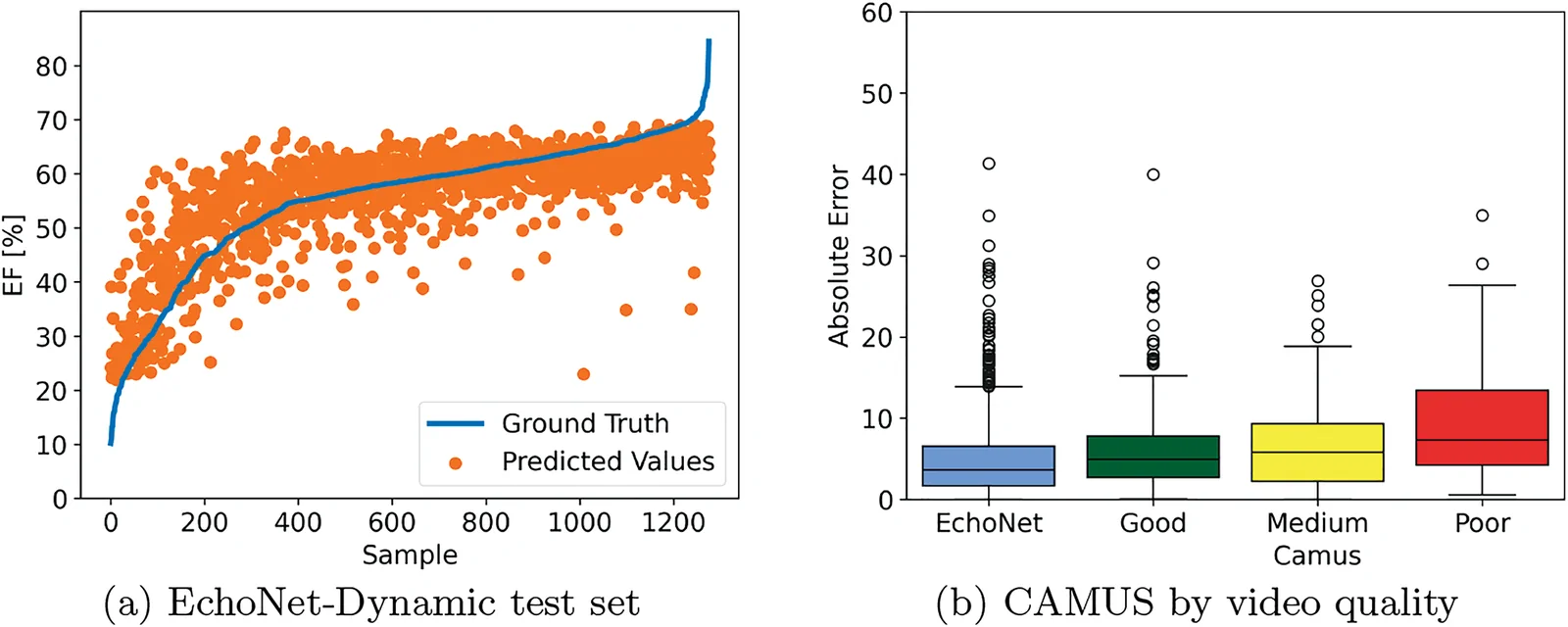
CAFEx EF prediction behavior. Panel (**a**) shows EchoNet-Dynamic test-set predictions sorted by ground-truth EF. Panel (**b**) shows absolute EF errors for the EchoNet-Dynamic test set and CAMUS good-, medium-, and poor-quality subsets. The model performs well for many mid-range EF values, but the quality-stratified CAMUS results show that poor-quality videos remain a limiting factor

The prediction curves in Fig. 8 provide a qualitative explanation for the improvement. CAFEx reduces the variance of the prediction errors and follows the mid-range EF distribution more closely than the feature-extraction baseline. The remaining failure mode is also visible: high EF values are still underestimated, and poor-quality CAMUS videos in Fig. 9 remain substantially more difficult than good- or medium-quality videos. Thus, the gains over the reproduced feature-extraction baseline are consistent but do not remove the need for image-quality assessment and uncertainty handling.

### Segmentation, phase dependence, and error patterns

**Fig. 10:**
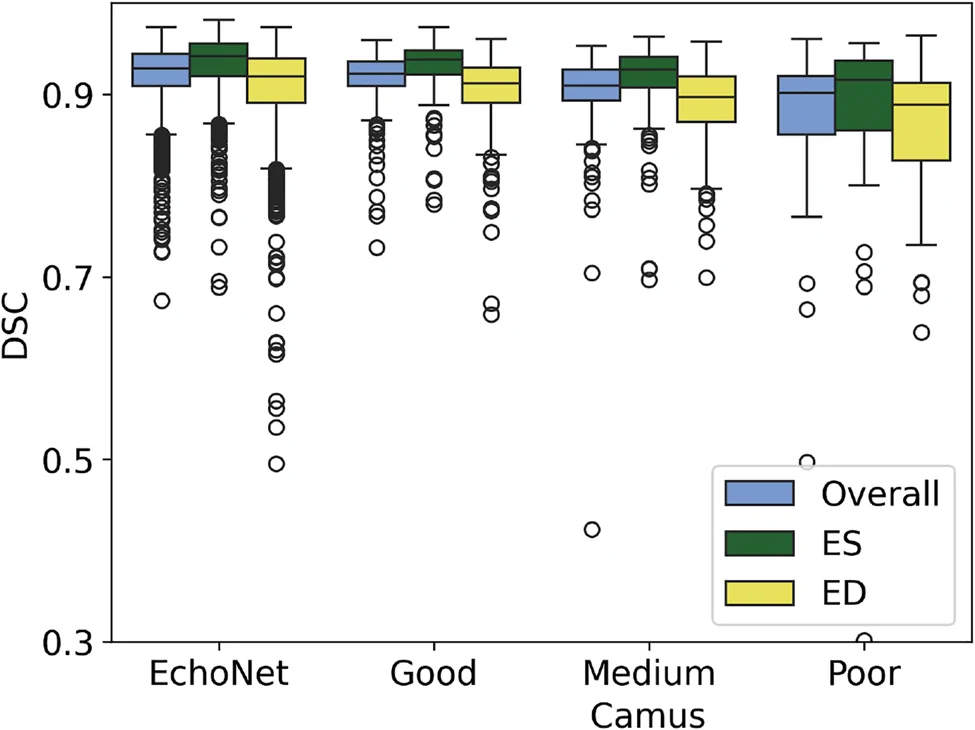
CAFEx segmentation Dice scores split by dataset, CAMUS video quality, and ED/ES frame type. ED frames are consistently more difficult than ES frames, and poor-quality CAMUS videos show the largest variance

Segmentation errors affect feature-based EF prediction because the geometric descriptors are computed from the predicted masks. Across models, ED frames are segmented less accurately than ES frames. This is clinically plausible: the LV cavity is larger at ED, the endocardial boundary can be more ambiguous, and errors in basal regions affect the derived long-axis and short-axis measurements. Figure 10 shows the CAFEx segmentation Dice distribution stratified by dataset, CAMUS quality, and ED/ES phase. Good- and medium-quality CAMUS videos preserve high segmentation performance, whereas poor-quality videos show larger variance.

The results also clarify the error propagation pathway. ED errors can have a large effect on EF because they alter the estimated end-diastolic volume. Poor-quality videos introduce a second pathway: when the endocardial boundary is not visible, the feature extractor may produce anatomically plausible but incorrect measurements. These errors are not uniformly distributed across the EF range. Extreme EF values, especially high values, are more likely to be pulled toward the center of the training distribution, which is consistent with regression-to-the-mean behavior. From a clinical perspective, this matters because extreme EF values are among the most relevant cases for diagnosis, therapy monitoring, and triage.

Taken together, the results suggest two empirical patterns in this benchmark. First, cross-dataset EF estimation appears substantially harder than in-distribution testing suggests, and the ranking of methods changes in the harmonized external evaluation. Second, an anatomical bottleneck combined with weak video-level EF supervision gives a more stable external predictor than direct video regression in this benchmark. The following discussion interprets these findings with respect to weak supervision, annotation effort, model capacity, and requirements for practical use.

## Discussion

### Principal findings and relation to external validation

This study indicates that automated EF estimation models can lose much of their predictive power when applied to a dataset different from the one used for training. The degradation is especially pronounced for direct EF regression models. While EchoCoTr and EchoGNN perform well on EchoNet-Dynamic, their CAMUS performance collapses to negative $R^2$ values. In contrast, the feature-based models retain positive explanatory power, and CAFEx achieves the best OOD EF prediction among the reproduced models. The key point is not that feature-based methods are preferable in all settings, but that their inductive bias changes the failure mode under dataset shift.

This behavior is consistent with the broader principle that external validation estimates a different quantity from internal validation. Internal validation measures interpolation within the source distribution. External validation measures transfer under changes in acquisition, patient selection, preprocessing, annotation convention, and target distribution. For annotation-efficient learning, the second quantity is especially important, because weak labels are often noisy and correlated with site-specific clinical workflows. Without external validation, a weakly supervised model may appear accurate while relying on non-transferable correlations.

The results therefore suggest the value of anatomical intermediate representations. EF is mechanically related to the change in ventricular volume between ED and ES. A model that first segments the left ventricle and then computes features from the mask sequence is forced to base its prediction on clinically meaningful geometry. This constraint does not guarantee perfect generalization, because segmentation itself can fail and geometric feature extraction can be noisy, but it appears to reduce the risk of learning non-transferable shortcuts. CAFEx improves on the reproduced feature-extraction baseline by strengthening the segmentation stage through contrastive pretraining and augmentation and by systematically testing feature and temporal-regression variants.

### Weak supervision and annotation effort

The benchmark can be interpreted through the amount and type of supervision used by each model family. Direct regressors require only video-level EF labels, which is attractive from an annotation perspective. However, their negative CAMUS $R^2$ values indicate that weak supervision alone is not sufficient for stable performance in this external benchmark. In contrast, segmentation-only models use stronger dense supervision and yield stable masks, but segmentation accuracy by itself does not solve EF regression. CAFEx combines the two: limited dense labels establish the anatomical coordinate system, while weak EF supervision trains the final prediction. In this benchmark, this combination is more stable on the harmonized external cohort than direct regression and more task-oriented than segmentation alone.

The results therefore do not imply that dense annotation is unnecessary in general. Rather, they suggest that dense annotation should be used strategically. A limited amount of dense annotation can impose anatomical structure on the representation, whereas the large-scale EF regression signal can remain weak and video-level. This is aligned with the practical distribution of labels in echocardiography archives: many studies contain report-derived EF values, but only a minority contain expert contours. The CAMUS performance of CAFEx suggests that this label configuration can be associated with improved recognition quality without scaling dense annotation proportionally with the number of videos in this setting.

From the perspective of methods for enhancing recognition quality with less annotation effort, the study provides an application-level example of three principles. First, dense annotation is used selectively to define an anatomical representation rather than exhaustively to label every video frame. Second, contrastive initialization exposes the segmentation backbone to image variation without requiring manual contours for every transformed view. Third, the final regressor is restricted to low-dimensional features related to ventricular mechanics. This interpretation is tied to the implemented CAFEx pipeline and its experimental behavior rather than to a generic semi-supervised learning schematic.

Although the experiments are implemented as a concrete EF-estimation pipeline, the training logic belongs to a broader weakly supervised learning taxonomy. The segmentation backbone receives strong supervision only for selected frames. Contrastive pretraining uses image augmentations and does not require manual contours. The EF regressor receives only the video-level target. Consequently, different parts of the model exploit different levels of annotation granularity. This mixed-supervision design is often more realistic than assuming a single label type for an entire dataset.

### Model capacity, inductive bias, and failure modes

The observed results can be understood as a trade-off between model capacity and inductive bias. Direct transformer and graph-based regressors have high representational capacity and can learn subtle temporal and spatial patterns from EchoNet-Dynamic. This capacity can be beneficial when the test set follows the same distribution, but it also permits the model to encode correlations that are not stable across datasets. Examples may include characteristic cropping, frame-number distributions, gain settings, or correlations between acquisition quality and EF in the source cohort. Because these variables are not causally tied to EF, a change in dataset can produce systematic prediction errors.

Segmentation-based feature pipelines reduce this risk by narrowing the information channel. Once the video is converted into a sequence of masks or geometric measurements, the regressor cannot directly access most scanner- and site-specific appearance features. This does not make the model immune to distribution shift, since segmentation itself can fail under poor image quality, but it changes the nature of the failure. The failure becomes more anatomical and therefore easier to inspect: a wrong EF prediction can often be traced to a poor mask, an incorrect apex, a basal contour error, or an implausible temporal sequence. Such error modes are more readily interpretable than opaque prediction drift in a direct video model.

However, a strong inductive bias can also limit performance. CAFEx may discard information that is useful for EF, such as subtle wall-motion patterns, regional contraction timing, or visual cues indicating foreshortening. This may explain why the best in-distribution direct model remains competitive on EchoNet-Dynamic. The central question is therefore not whether direct or anatomical models are preferable in all settings, but which representation is preferable when annotation effort and external generalization are considered simultaneously. The evidence in this study favors anatomical feature learning in the evaluated external weakly supervised setting, while future systems may benefit from combining anatomical features with uncertainty-aware motion representations.

The consistent segmentation gap between ED and ES frames deserves further study. EF estimation depends on the relationship between EDV and ESV; therefore, ED segmentation errors can propagate strongly into the final EF estimate. Improving ED segmentation may require phase-aware losses, temporal consistency constraints, or explicit modeling of the mitral valve plane and apex. Transformer segmentation backbones with stronger augmentation may also help, as augmentation improved the simpler Swin configuration substantially.

### Preprocessing, harmonization, and reproducibility

The preprocessing pipeline has a direct effect on how the results should be read. Cropping and resizing CAMUS to *112×112* pixels imposes the same input resolution used by EchoNet-Dynamic models, but it also discards high-frequency boundary information that might be useful for segmentation. CAMUS frames have substantially higher and variable native spatial resolution, so this experiment evaluates a harmonized downsampled benchmark rather than clinically realistic native-resolution segmentation. At *112×112* pixels, a one-pixel boundary displacement can represent a meaningful anatomical shift relative to the LV cavity, especially near the apex or mitral-valve plane. Because EF is derived from the difference between EDV and ESV, small systematic boundary errors can be amplified in the final EF estimate. Thus, the fact that CAFEx retains a Dice score above 90% under this transformation suggests robustness to this forced low-resolution setting, but it should not be read as evidence that the same boundary accuracy would hold in native-resolution clinical deployment. The residual EF error also indicates that good low-resolution segmentation is necessary but not sufficient for accurate EF estimation under the evaluated conditions.

The EF recalculation step is equally important. CAMUS provides biplane EF, while EchoNet-Dynamic provides released clinical EF labels whose exact public measurement procedure is not explicitly specified. If the original CAMUS EF values were used directly, the evaluation would mix image-domain transfer with a known biplane-versus-single-plane target mismatch. Recalculating CAMUS EF from the apical four-chamber masks does not make the target identical to EchoNet-Dynamic, because differences in clinical measurement procedure, contouring rules, and phase selection may remain. Nevertheless, it defines the CAMUS endpoint used in this benchmark and reduces a major avoidable source of mismatch. In clinical terms, the experiment evaluates whether the model transfers across cohorts under an explicitly harmonized single-plane external target, not whether a model can emulate the original CAMUS biplane reference.

The conversion from dense CAMUS labels to EchoNet-compatible masks also illustrates a general difficulty in annotation-efficient medical imaging. Public datasets frequently differ not only in image appearance but also in what is annotated. One dataset may provide sparse landmarks, another dense masks, and another only report-level labels. A practical learning system must therefore operate across heterogeneous annotation regimes. CAFEx uses this heterogeneity constructively: dense labels are used where available to train the anatomical component, while report-level EF values supervise the final regression. This reflects a common scenario in large clinical archives, in which not every patient has the same annotation granularity.

Reproducibility in cross-dataset EF estimation requires more than reporting model architecture and optimizer settings. The preprocessing choices, mask conversion rules, EF recalculation method, train/test splits, frame sampling, and quality stratification all influence the outcome. For this reason, the paper reports not only final EF metrics but also segmentation Dice scores, reproduction checks, ablations, and quality-specific error analyses. These components help identify whether a performance difference arises from the segmentation backbone, the feature extractor, the temporal regressor, or the target harmonization.

The use of both MAE and $R^2$ is also important. MAE is clinically intuitive because it is measured in EF percentage points. However, MAE alone can be misleading when EF distributions differ across datasets. A model that predicts values near the source-domain mean may have an acceptable MAE on a narrow target distribution but explain little of the target variance. Negative $R^2$ values in the direct-regression baselines are therefore not a technical artifact; they indicate that the predictions are worse than a target-mean predictor on the external cohort. Reporting both metrics is necessary for a fair comparison under distribution shift [34, 35].

### Limitations and future work

Several methodological limitations should be acknowledged. The study uses CAMUS as an OOD benchmark after substantial preprocessing. Although this harmonization is necessary for this controlled comparison, it does not fully reproduce a real application-to-clinical-workflows scenario, where models may encounter arbitrary view quality, scanner metadata differences, incomplete cardiac cycles, and non-apical-four-chamber views. The CAMUS target was recalculated to approximate a single-plane apical-four-chamber EF endpoint, but residual differences from EchoNet-Dynamic labels may remain because the exact released EchoNet-Dynamic EF measurement procedure is not specified in the public documentation. Furthermore, not all baselines could be reproduced exactly, and some were adapted because publicly available implementations differed from the experimental protocol. The quantitative tables report the reproduced single-run benchmark values; standard deviations across repeated training runs or random seeds were not recorded for all baselines and therefore are not reported. Per-video prediction exports and repeated-seed logs were also not retained uniformly for all reproduced baselines, so predicted-versus-ground-truth scatter plots, Bland–Altman plots, and mean *±* standard deviation summaries could not be generated consistently across methods in this revision. The goal of the benchmark is therefore comparative insight under a consistent setup, not a final leaderboard.

The quality-stratified evaluation also shows that poor ultrasound quality is not a rare exception in clinical practice. A model that performs well only on high-quality images may still be useful in restricted decision-support settings, but it requires a quality-control mechanism and a conservative use policy. The present results suggest that weakly supervised EF estimation should be paired with uncertainty estimation, image-quality assessment, or rejection rules. Such mechanisms are not implemented here, but the observed error structure provides motivation for them. Clinically, the current CAFEx performance should therefore be interpreted as research evidence for a more robust cross-dataset representation, not as sufficient validation for autonomous EF measurement. The negative $R^2$ values in poor-quality CAMUS videos and the regression-to-the-mean behavior at extreme EF values are important failure modes. At the present performance level, cases with poor image quality, out-of-range anatomy, or EF values near therapeutic decision thresholds would require human review and additional calibration rather than automatic acceptance of the model output.

Future work should extend this evaluation to additional external datasets and prospective clinical cohorts. CAFEx could be improved by tighter integration of the contrastively pretrained segmentation model, joint optimization of segmentation and EF regression, uncertainty estimation, and richer features that combine short-axis lengths, areas, contour coordinates, apex and mitral valve positions, and temporal derivatives. Sequence models that handle variable-length input through masking rather than padding may further improve stability in similar evaluations. Finally, clinical translation would require calibration studies, reader studies, quality control, privacy-preserving implementation, and regulatory validation.

## Conclusion

Cross-dataset evaluation and annotation efficiency are central challenges for automated EF estimation from echocardiography. In a train-on-EchoNet/test-on-CAMUS evaluation, several high-performing in-distribution models degraded substantially, especially direct video-regression models trained from weak video-level labels alone. CAFEx was designed to examine whether limited anatomical supervision, contrastive-augmented left ventricular segmentation, explicit temporal feature extraction, and weakly supervised EF regression can provide a more stable alternative in this setting. The method obtains high segmentation performance on both EchoNet-Dynamic and CAMUS and achieves the highest CAMUS EF prediction performance among the reproduced baselines in this benchmark, with $R^2=0.54$ and MAE of 6.70%. These findings suggest that anatomically grounded pipelines remain relevant for medical imaging models evaluated beyond their source dataset and that future EF estimation studies would benefit from reporting external validation under controlled and clinically motivated distribution shifts.

## Data Availability

The datasets analyzed during the current study are publicly available in the EchoNet-Dynamic repository at https://stanfordaimi.azurewebsites.net/datasets/834e1cd1-92f7-4268-9daa-d359198b310a and in the CAMUS repository at https://www.creatis.insa-lyon.fr/Challenge/camus/databases.html. The code and training scripts used for the experiments reported in this manuscript will be made publicly available upon publication at https://github.com/Adrian398/Ejection-Fraction-Estimation.

## Abbreviations

A4C: Apical four-chamber
CAFEx: Contrastive-augmented feature extraction
CAMUS: Cardiac Acquisitions for Multi-structure Ultrasound Segmentation
ED: End-diastole
EDV: End-diastolic volume
EF: Ejection fraction
ES: End-systole
ESV: End-systolic volume
LV: Left ventricle
MAE: Mean absolute error
OOD: Out-of-distribution.
